# Mathematical modelling of post-filter ionized calcium during citrate anticoagulated continuous renal replacement therapy

**DOI:** 10.1371/journal.pone.0247477

**Published:** 2021-02-25

**Authors:** Innas Forsal, Anders Nilsson, Mikael Bodelsson, Anders Wieslander, Marcus Broman

**Affiliations:** 1 Skåne University Hospital, Lund, Sweden; 2 Baxter International Inc., Lund, Sweden; Mathematical Institute, HUNGARY

## Abstract

**Background/Aims:**

Post-filter ionized calcium (iCa) measured on a blood gas analyzer (BGA) during regional citrate anticoagulated continuous renal replacement therapy (CRRT) are needed to control the regime. This increases the workload and requires attention including interpretation of blood analyses. Two algorithms were developed to calculate the post-filter iCa instead. The first algorithm used measured systemic total calcium and the second used a selected set of values from an initial blood gas sample as input.

**Methods:**

Calculated post-filter iCa values were compared to real blood gas analyses. 57 patients treated at the intensive care unit at Skåne University Hospital in Lund during 2010–2017 were included after applying inclusion and exclusion criteria. Clinical and machine parameters were collected from the electronic medical records. Non-quality checked data contained 1240 measurements and quality checked data contained 1034 measurements.

**Results:**

The first algorithm using measured systemic total calcium resulted in slightly better precision and trueness with an average difference between the predicted and measured post-filter iCa concentration of 0.0185±0.0453 mmol/L for quality checked data, p<0.001. Neither algorithm could detect all instances requiring intervention.

**Conclusion:**

The algorithms were able to estimate in range postfilter iCa values with great trueness and precision. However, they had some difficulties to estimate out-of-range postfilter iCa values. More work is needed to improve the algorithms especially in their citrate-modelling.

## Introduction

Continuous renal replacement therapy (CRRT) is the first choice in critically ill patients with acute kidney injury (AKI) and hemodynamic instability. Anticoagulation is needed during CRRT, because blood comes into contact with tubing and filter membranes, which initiates a cascade of reactions leading to clotting and reduced filter life span [1, 2]. Systemic heparin has for a long time been the most common anticoagulant used [3, 4] but methods that anticoagulate explicitly the dialysis circuit have been developed. Regional citrate anticoagulation (RCA) is today a strong contender to heparin and has become the first choice [5, 6]. Citrate can however lead to systemic hypocalcemia, metabolic alkalosis and in some cases to direct citrate toxicity, and requires a meticulous monitoring [7, 8]. These disturbances have been reduced by monitoring of laboratory values and usage of structured protocols, such as the Flexitrate protocol, which is based on regular monitoring among others systemic ionized calcium (iCa), post-filter iCa and systemic total calcium [4, 8]. Post-filter iCa indicates the efficiency of the anticoagulation in the filter. Together with systemic iCa it ensures that the filter is fully anticoagulated and that the patient at the same time stays normocalcemic [9–12].

Several steps have been taken to achieve automation of RCA during CRRT by using RCA software, which calculates the loss of calcium over the filter which is matched and compensated by adjusting intravenous calcium substitution. This software is based on machine data, dialyzer properties and a dialyzer transport model, which calculates the losses of free calcium over the dialyzer into the effluent.

The algorithms presented in this study are based on machine data, dialyzer properties, but includes a dialyzer transport model and a blood model that takes into account changes in blood chemistry due to the dialysis like addition of solutes from the solutions used and citrate which chelates ionized calcium. The setup is illustrated in a S1 Fig.

Several studies have tried to simplify RCA treatment in intermittent hemodialysis (IHD) by using algorithms, which use both pre- and post-filter iCa indicators of the circuit anticoagulation [13].

Our present study is the first regarding CRRT. In order to replace (reduce) sampling and measuring of post-filter iCa we developed two algorithms that use data from the CRRT machine and patient data in the form of systemic total calcium, which can either originate from the laboratory (first algorithm) or be calculated by using data from an initial blood gas analysis (second algorithm). Both algorithms include modelling of patient citrate concentrations, i.e. the algorithms calculate the amount of citrate that will reach the patient when using a certain citrate solution during CRRT.

The aim of this study was to evaluate the trueness and precision of the algorithms based on comparing the calculated post-filter iCa to the real measured blood gas analyzer (BGA) value.

## Methods

### Mathematical and chemical assumptions behind the algorithms

Calculation of post-dialyzer iCa requires a model simulating the interactions of calcium with other substances in the blood. These interactions are defined by chemical reactions. The outcome is determined by the local concentration of the reactants, chemical equilibrium constants and the total amounts of calcium and other participating substances. The total of a certain substance is the sum of all its forms. The local concentrations depend on the composition of the blood that is pumped out of the patient and the fluids that are mixed into the extracorporeal circuit and transported over the dialyzer membrane.

The blood-chemistry includes free and bound calcium. In this model calcium bound to citrate, albumin, bicarbonate, phosphate, and carbonate is handled. Magnesium, sodium and hydrogen are competing to calcium regarding binding.

The patient’s systemic concentration of calcium is measured either as a total concentration or as ionized (free) concentration. The other modelled substances are assumed to be at a constant level over time with the exception of citrate which is modelled over treatment time based on the supplied citrate for anticoagulation, which imposes a strong effect on the balance between free and bound calcium, and is therefore important to the model. The body always tries to keep the ionized calcium as constant as possible, and it should be noted that normal equilibrium of calcium concentration is not reached during RCA CRRT due to the constant addition of citrate and the removal of substances and addition of solutions during the dialysis itself. Addition of citrate into the dialysis solutions causes the equilibrium between bound and free calcium to change.

The mass flow of each substance into the mixing point is simulated and gives the final concentration. Calculation of post-filter calcium is dependent on the amount of the total calcium which is free for transport over the dialyzer and protein-bound calcium which cannot pass the dialyzer. A big portion of the free calcium will bind citrate and citrate-calcium complexes and will be lost through the dialyzer. In order to be able to derive the clearance, i.e. how much of a solute will be removed, the flow rates and the concentrations of the solute at the inlets of the dialyzer must be known.

When citrate binds to calcium in the blood it leads to calcium being released from albumin in an attempt to maintain the equilibrium between free ionized and protein bound calcium. This shift must be taken into account during the clearance of ionized calcium in the extracorporeal circuit.

Each reaction modelled is quantified by its equilibrium constant (*K*_*C*_) giving one equation of type $\frac{\left[AB\right]}{\left[A]*[B\right]}=K_{C}$ where the complex AB is formed from A and B [14] and denotes concentration. Twenty-six equilibrium equations are handled and eleven equations for totals of Ca and electroneutrality give a non-linear system of 37 equations. Numerical solving of these equations gives free Ca as a function of the totals, which could be denoted as a function $\left(Ca^{2^{+}}Ca^{2^{+}}=Ca_{t_{o_{t}}},Mg_{t_{o_{t}}},Na_{t_{o_{t}}},Cl_{t_{o_{t}}},K_{t_{o_{t}}},HCO_{3_{t_{o_{t}}}},HPO_{4_{t_{o_{t}}}},SO_{4_{t_{o_{t}}}},Cit_{t_{o_{t}}},Acet_{t_{o_{t}}},Lact_{t_{o_{t}}},Alb_{t_{o_{t}}}\right)$. Full solving of this function requires a rather high computing capacity.

In order to make it easier to implement in a dialysis machine we need to simplify the function $Ca^{2^{+}}$. Computer simulations show that the solution is dependent to a lower degree to most of the arguments, including albumin. The highest dependence is for totals of calcium and citrate, and thus a linearization Ca^2+^_lin_ for these two variables was carried out. Due to the number of equations a linearization was preformed to simplify the calculations, but also to make the algorithms more suitable for CRRT machines with limited processing capacity.

### The main equation to calculate post-filter iCa

Eq 1 is valid for every point in the solution, while Eqs 2–6 are valid for post-filter iCa calculation.

$$Ca_{lin}^{2+}(Ca_{tot},Cit_{tot})=Ca^{2+}(Ca_{tot,0},Cit_{tot,0})+\frac{\partial Ca^{2+}}{\partial Ca_{tot}}(Ca_{tot,0},Cit_{tot,0})*(Ca_{tot}-Cit_{tot,0})+\frac{\partial Ca^{2+}}{\partial Ca_{tot}}(Ca_{tot,0},Ca_{tot,0})*(Cit_{tot}-Cit_{tot,0})$$ (1)

*Ca*_*tot*,0_ and *Cit*_*tot*,0_ are the center of linearization. *F*_*CAL*_ is the fraction of patient plasma total calcium that is made available in plasma water for transfer through the dialyzer membrane after citrate infusion, this is a constant that can be set to 0.98. *Ca*_*sys*_ is the total calcium concentration in the blood of the patient, given by laboratory analysis or conversion from values from the blood gas analyzer, the difference between algorithm 1 and 2 is in *Ca*_*sys*_. In algorithm 1 it comes from the laboratory meanwhile for algorithm 2 it comes from conversion of values from a blood gas analyzer, i.e. ionized total calcium.

$$Ca_{pw}=(Q_{P}-K_{Ca}*F_{CAL}*F_{DIL}*Ca_{sys})*(\frac{1}{Qpw_{inlet}-Q_{fil}})$$ (2)

K_Ca_ is the clearance of calcium in the dialyzer. The different parts used for Eq 2 are defined in Eqs 3–10. The different flows used in the equations are plasma flow (Q_*P*_), blood flow (Q_*B*_), dialysate flow (Q_*D*_), effluent flow (Q_*E*_), flow of replacement fluid downstream of dialyzer (Q_PRE_) and flow of citrate-containing fluid (Q_PBP_). Hematocrit (Hct) is also used.

The plasma flow rate (*Q*_*P*_) can be written as:

$$Q_{P}=(1-Hct)*Q_{B}*60$$ (3)

The filter clearance for calcium (K_Ca_) is defined as below:

$$K_{Ca}=\frac{Qpw_{inlet}*Q_{D}-f*(Qpw_{inlet}-Q_{fil})*(Q_{D}+Q_{fil})}{Q_{D}-f*(Qpw_{inlet}-Q_{fil})}$$ (4)

where

$${\left(\frac{Qpw_{inlet}-Q_{fil}}{Qpw_{inlet}}*\frac{Q_{D}+Q_{fil}}{Q_{p}}\right)}^{\frac{1}{\gamma }}$$ (5)

and

$$\gamma =e^{\frac{Q_{fil}}{K_{o}A}}-1$$ (6)

K_o_A defines membrane specific properties and the value is unique for the dialyzer used.

The plasma water flow rate (Qpw_inlet_) at the filter inlet can be written as:

$$Qpw_{inlet}=Qpw+Q_{PBP}+Q_{PRE}$$ (7)

Plasma water flow rate (Qpw), where fpw is is the plasma water fraction:

$$Qpw=(1-Hct)*fpw*Q_{B}*60$$ (8)

The dilution ratio from the plasma water (F_DIL_) reaching the dialyzer:

$$F_{DIL}=\frac{Qpw}{Qpw_{inlet}}$$ (9)

Filtration flow rate (Q_fil_) is defined as:

$$Q_{fil}=Q_{E}-Q_{D}$$ (10)

The extended model is configured to handle 26 chemical reactions, and after adding equations for totals of involved substances including calcium and electroneutrality, the extended model results in a non-linear system of equations as mentioned above.

The extended model will also lead to a formula to calculate the total calcium after the dialyzer (Ca_PD_) (see Eq 11). To be able to get *Ca*_*PD*_ calculation of the total calcium in plasma water (Ca_pw_) is required (see Eq 2).

The formula for total calcium after the dialyzer (Ca_PD_) can be written:

$$Ca_{PD}=Ca_{pw}*fpw_{PD}$$ (11)

where the plasma water fraction post-dialyzer (fpw_PD_) is defined as below.

fpw is the plasma water fraction and is dimensionless. Q_fil_ is defined in Eq 10:

$$fpw_{PD}=1-\frac{Q_{B}*60*(1-Hct)}{\left(1-Hct_{PD})*(60*Q_{B}+Q_{PBP}+Q_{PRE}-Q_{fil}*(1-fpw)\right)}$$ (12)

fpw_PD_ uses the hematocrit post-dialyzer (Hct_PD_):

$$Hct_{PD}=Q_{B}*60*\frac{Hct}{Q_{B}*60+Q_{PBP}+Q_{PRE}-Q_{fil}}$$ (13)

The model will also calculate the total citrate after the dialyzer, i.e. Cit_PD_, by considering mass flow balance and losses in the dialyzer:

$$Cit_{pw}=(D_{cit}*Q_{B}*60)+(Cit_{sys}*Q_{P})*(1-\frac{K_{cit}}{Qpw_{inlet}})*(\frac{1}{Qpw_{inlet}-Q_{fil}})$$ (14)

where the citrate dose (D_cit_) is defied below and where Cit_PBP_ is the citrate concentration of the solution used. D_cit_ can be given from the machine settings or be calculated from the formula below:

$$D_{cit}=\frac{Cit_{PBP}*Q_{PBP}}{Q_{B}*60}$$ (15)

The total citrate in the plasma post-dialyzer (Cit_PD_) is then defined as:

$$Cit_{PD}=Cit_{pw}*fpw_{PD}$$ (16)

By combining the formulas for removal of total calcium and citrate with the combination of the citrate metabolism one will be able to calculate post-filter iCa.

### Solute transport over the dialyzer

The algorithms for dialyzer transport were based on the previous work done by Sternby et.al. where they have mathematically modelled the diffusive-convective mass transfer rates in dialyzers for solutes present on both sides of the membrane for uncharged solutes. In the report the mass transport rate is assumed to be linear in both inlet concentrations, since the driving forces for diffusion and convection are linear [2]. In our case with charged substances, including the electrical forces will make the transport non-linear, but for simplicity we ignore this effect. The equations were modified to fit assumptions needed to be done for calculating the removal of ionized calcium over the dialyzer.

The filter clearance (K) is defined as:

$$K=\frac{Qpw_{inlet}*Q_{D}-f*(Qpw_{inlet}-Q_{fil})*(Q_{D}+Q_{fil})}{Q_{D}-f*(Qpw_{inlet}-Q_{fil})}$$ (17)

where

$$f={\left(\frac{Qpw_{inlet}-Q_{fil}}{Qpw_{inlet}}*\frac{Q_{D}+Q_{fil}}{Q_{D}}\right)}^{1/\gamma }$$ (18)

and

$$\gamma =e^{\frac{Q_{fil}}{K_{o}A}}-1$$ (19)

k_o_A is dependent on membrane specific properties. To be able to calculate clearance for charged particles consideration of electroneutrality and complex binding is required. Electroneutrality is maintained through every point along the dialyzer and needs to be handled in the local transport across the membrane. Electroneutrality can be maintained by introducing membrane potential, which is a potential that is present due to an existing difference in the transport of different ions causing a polarization across the membrane. The value of the membrane potential will determine electroneutrality.

The removal rate *J* can be written as:

$$J=K_{b}*c_{bi}-K_{d}*c_{di}$$ (20)

To be able to handle the fact that the membrane potential varies along the dialyzer the dialyzer can be divided into a number of subdialyzers, ten is often enough, along its length. In this way the transport can be calculated separately in each section of the dialyzer. By splitting the dialyzer into different sections handling of complex binding of specific ions to each other and other chemical equilibrium will be possible [2]. All this is done iteratively and the result can be used for the linearization.

### Formulas for predicting citrate in patients

The citrate load (G_met_) that is the amount of citrate that enters the patient´s body is given by:

$$G_{met}=0.0167*PatWeight$$ (21)

The plasma clearance of citrate by the filter is given below. The assumption is that K_Ca_ = K_citrate_, i.e. that the clearance of citrate over the filter is the same as for calcium and for the whole equation see Eqs 4–6:

$$K_{Dialyzer}=\frac{K_{citrate}}{1000}*(\frac{Q_{pw}}{Q_{pw}+Q_{rep}+Q_{PBP}})$$ (22)

The citrate load (J_citload_) in the patient is defined as:

$$J_{citload}=D_{cit}*Q_{B}*60*10^{-3}*(1-\frac{\frac{K_{citrate}}{1000}}{Q_{pw}+Q_{rep}+Q_{PBP}})$$ (23)

Volume of citrate distribution (V_*d*_):

$$V_{d}=\frac{29}{72}*PatientWeight$$ (24)

Clearance of citrate in the body (K_patient_):

$$K_{patient}=7.79*PatientWeight*60*10^{-3}$$ (25)

The systemic citrate (C_sys_) in the patient is given by Eq 26. It uses K_dialyzer_ which is the plasma clearance of citrate through the dialyzer and C_0_ which is the concentration of the plasma citrate at the time zero:

$$C_{sys}=C_{0}*e^{\left(-(K_{dialyzer}+K_{patient})*(\frac{t}{V_{d}})\right)}+(\frac{G_{met}+J_{citload}}{K_{dialyzer}+K_{patient}})*(1-e^{\left(-(K_{dialyzer}+K_{patient})*(\frac{t}{V})\right)})$$ (26)

Normally the systemic citrate (C_sys_) in the patient is low, 0.13 mmol/l, and during RCA CRRT the systemic citrate will increase to between 0.3–0.6 mmol/l or even higher, since some of the citrate given during the treatment will not be removed through the dialyzer and will be returned to the patient [8, 10]. This increase in systemic citrate is taken care of by the algorithm (Eqs 23–26) to calculate systemic citrate in patient [10, 11].

### Conversion of ionized calcium to total systemic calcium

The notations for the input values below are defined in the section *Notations used*.

*iCa*_*ref*_ = 1.25
*iCit* = 0.020
*iP* = 0.465
*K*_*CaB*_ = 10^0.6^
*K*_*CaCit*_ = 10^3.364^
*K*_*CaCO3*_ = 10^7.18^
*K*_*CaP*_ = 10^1.8^
*K*_*CaCit*2_ = 10^4.964^

Number of sites that are occupied by calcium on a protein (nCa_ref_) is defined as:

$$nCa_{ref}=1.58+0.907*(pH-7,4)$$ (27)

Plasma clearance of calcium (K_Ca_) is defined as:

$$nCa_{ref}=1.58+0.907*(pH-7,4)$$ (28)

Total systemic calcium in the patient (totCa_sys_):

$$totCa_{sys}=iCa*(1+12*\frac{Albumin}{K_{Ca}+iCa})+\frac{Bicarbonate}{1000}*K_{CaB}+\frac{Bicarbonate}{\frac{1000}{\frac{10^{-pH}}{K_{CaCO3}}}}+iP*K_{CaP}*1000+\frac{iCit*K_{CaCit}}{1000}+\frac{iCit*\frac{iCit*K_{CaCit2}}{1000}}{1000}$$ (29)

To be able to use data from the BGA to calculate total systemic calcium a conversion formula was developed. The conversion formula was based on bicarbonate, systemic iCa, pH and albumin (fixed value can be used or value from laboratory when analyzed) to calculate systemic total calcium. The calculation of total systemic calcium from ionized calcium is dependent on several factors on which, the balance between soluble and bound calcium will be affected. The different constants used in 29 are taken from literature. Albumin concentration will result in a shift in the ratio of free and bound calcium. Albumin has 12 binding sites that can be occupied by free calcium and this number (nCa_ref_) is dependent on pH. The equilibrium association constant for calcium $\left(K_{C_{a}}\right)$ is a mass law that predicts systemic total systemic calcium with the free calcium and number of sites that are occupied by calcium on a protein (nCa_ref_). The equilibrium association constants for other complexes, such as citrate and calcium have been reported in literature. The pH dependency can be removed if (*nCa*_*ref*_) is set as a constant. Free calcium is not only able to create complexes with albumin but also bicarbonate, citrate and phosphate, which needs to be taken into account. By calculating how free calcium can be bound it is possible to predict the total systemic calcium (totCa_sys_, 29) [14].

### The two algorithms

Two separate algorithms to predict postfilter iCa were constructed and the main difference was in the calcium input (Table 1). For Algorithm 1 the value Ca_tot_ is taken from the measurements from the laboratory and what the script does is take the closest value and newest measurement, if there is no new measurement old values will be reused. For Algorithm 2 we use a conversion formula to take iCa values from the BGA and recalculate it to *Ca*_*tot*_.

$$Postfilter\;iCa=\beta_{0}+\beta_{1}*(Ca_{PD}-Ca_{ref})+\beta_{2}*(Cit_{PD}-iCit)$$ (30)

where β_0_ and β_1_ and β_2_ are constants given by the linearization of the full model.

**Table 1:** Algorithm 1 used systemic total calcium and Algorithm 2 used an initial blood gas as input.

	Algorithm 1	Algorithm 2
**Patient parameter inputs**	The most recent measured systemic total calcium value from hospital laboratory	Systemic iCa from BGA
		pH from BGA
		Bicarbonate from BGA
		Albumin (fixed or from laboratory)
**Machine parameter inputs**	Blood flow	
	Dialysis fluid flow	
	Post-filter replacement fluid flow	
	Calcium replacement flow	
	Patient fluid removal rate	
	Pre-filter replacement fluid flow	
	Citrate dose	
	Composition of solutions used (i.e. concentration of calcium, bicarbonate, citrate, hydrogen phosphate, sodium, magnesium, potassium)	
	Filter elapsed treatment duration (blood pump running time)	

The setup with two algorithms with different input was to evaluate how altered input could influence the accuracy of the output; the postfilter ionized calcium. The algorithms were executed in MATLAB (MathWorks^®^).

### Study cohort

Data was extracted from the electronic medical record (Philips ICCA system) of 120 patients during years 2010–2017 at the Intensive Care Unit at Skåne University Hospital, Lund, Sweden. Of the 120 patients identified, 57 patients were finally analyzed.

The inclusion criteria were: (1) citrate as the only anticoagulation or in combination with other anticoagulation types; (2) filter information available; (3) systemic total calcium available; (4) post-filter iCa available. Exclusion criteria were: (1) insufficient data; (2) the patient was included in other studies.

### Ethical approval

The Regional Ethics Board of southern Sweden approved the study (Dnr 2017/618).

### Quality checked measured values

In the post-filter iCa measurements from the blood gas analyzer outliers were present, i.e. values that tended to occur out of range defined by the following criteria; (1) post-filter iCa above 1.0 mmol/l; (2) a single value that would have generated an intervention spontaneously reverting to normal without intervention; (3) single value that resulted in an intervention, but where the next value within 30 min resulted in a reversion to the previous setting.

Different scenarios can cause values out of range; wrong value inserted into wrong position in the electronic medical record (EMR), sampling error, measurement error etc. An outlier can be detected by the physician and overlooked, go totally undetected or get accounted for and thus alter the treatment in an unwanted way.

**Fig 1:**
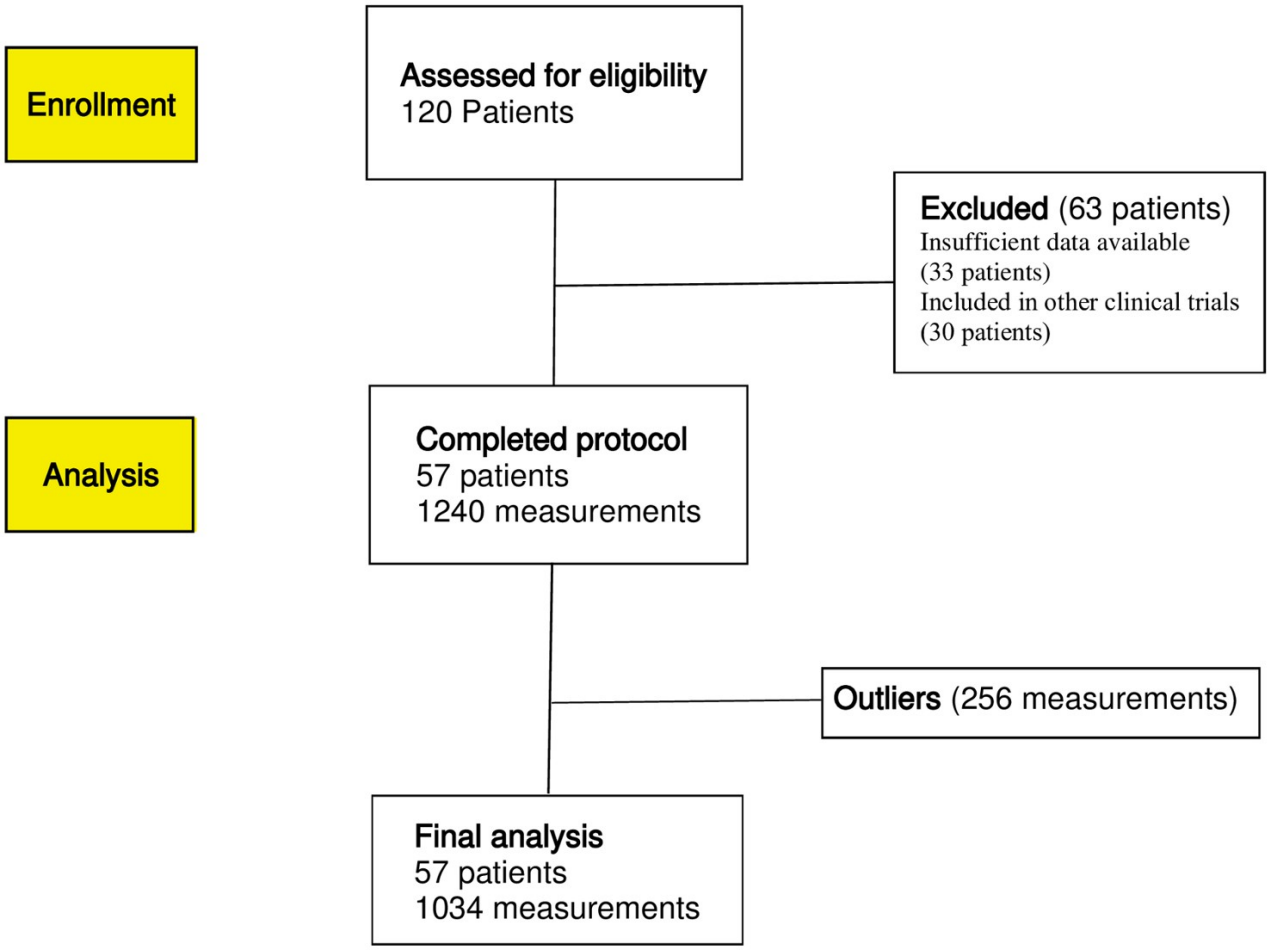
Flow diagram for inclusion and exclusion criteria for patient enrollment and measurement points analyzed.

The mathematical modeling was applied on the quality checked extracted patient data but also on the complete data set. Detailed overview of exclusions and inclusions made for patients and specific measurement is shown in Fig 1.

### Device settings

Prismaflex CRRT machine and ST-150 filters were used. The modalities were continuous venovenous hemodiafiltration (CVVHDF) in 56/57 patients and continuous venovenous hemodialysis (CVVHD) in 1/57 patients.

### Treatment data

Biochemistry and CRRT patient data were extracted and sorted to match flows according to timestamp.

## Results

Algorithm 1’s (input systemic total Ca) deviation from BGA was 0.0079 ±0.0709 mmol/L with outliers included and 0.0185 ±0.0453 mmol/L without, while Algorithm 2’s (input an initial BGA analysis) deviation was -0.0351 ±0.0727 mmol/L with outliers and -0.0249 ±0.0468 mmol/L without (Table 2). Thus, Algorithm 1 resulted in a better trueness (lower mean value, without outliers) and a better precision (lower standard deviation, without outliers), p<0.001.

**Table 2:** Mean and standard deviation for the modelled data compared to the measured value on BGA. Complete data set and quality checked data set included 1290 and 1034 individual measurements respectively in 57 patients. The number measurements for each patient could range from 3 unique post-filter iCa measurements to over 20.

	Algorithm 1	Algorithm 2
**Mean of complete data set [mmol/l]**	0.0079	-0.0351
**Mean of quality checked data set [mmol/l]**	0.0185	-0.0249
**Standard deviation (SD) of complete data set [mmol/l]**	0.0709	0.0727
**Standard deviation (SD) of quality checked data set [mmol/l]**	0.0453	0.0468

**Fig 2:**
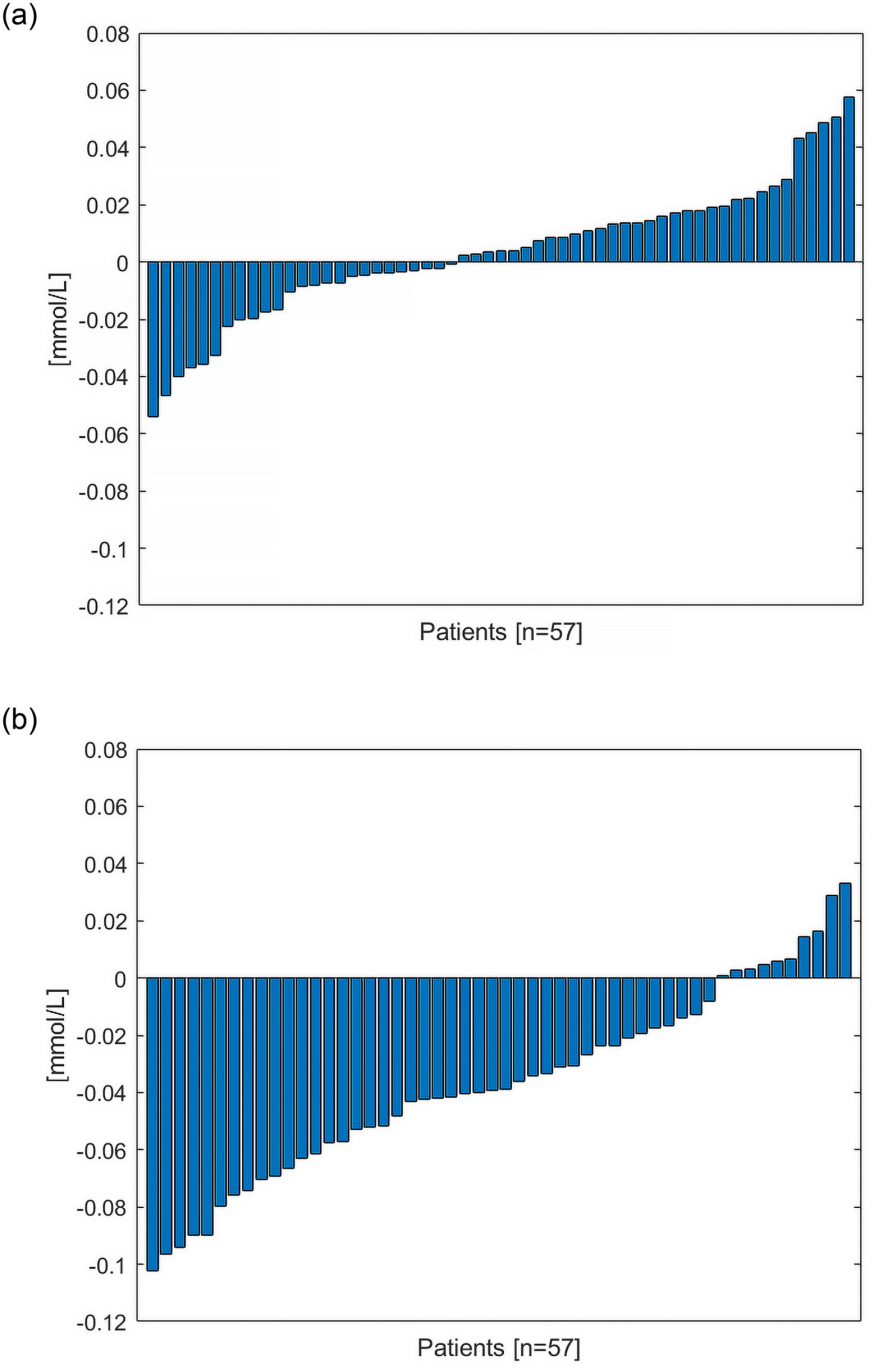
Average differences between measured post-filter iCa and calculated post-filter iCa for quality checked data for Algorithm 1 (2a) and Algorithm 2 (2b). There are instances when the algorithms are returning values both higher and lower to the BGA. Algorithm 2 has a tendency to underestimate postfilter iCa, while Algorithm 1 does not seem to have the same tendency.

**Fig 3:**
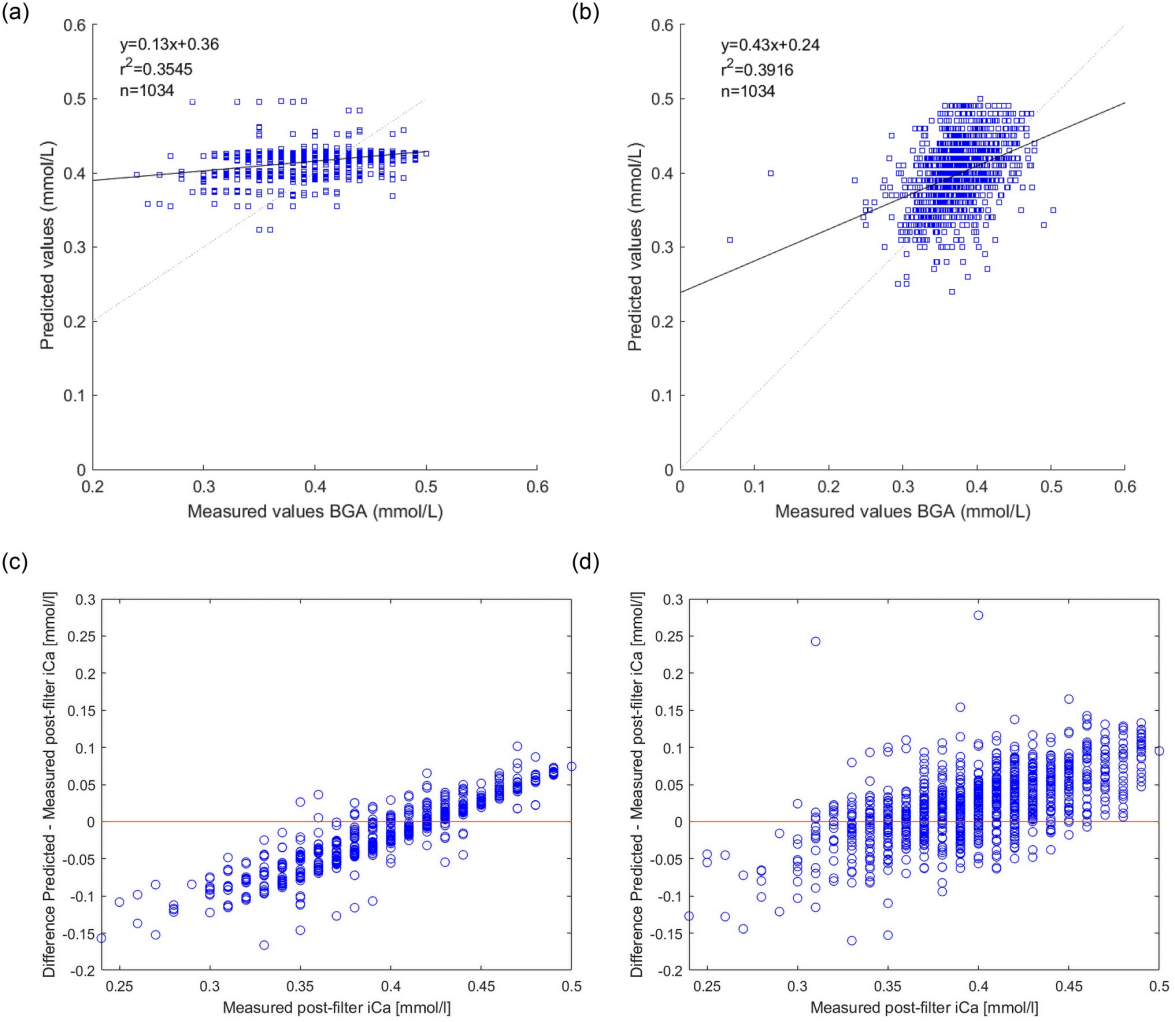
Regression plots for Algorithm 1 (3a) and Algorithm 2 (3b). r^2^ is the Pearson R-value squared, SSE is the sum of squared error. The equation for the regression line is also calculated and shown as a straight black line. The ideal equation would be y = x and shown as a dotted black line. The differences between the calculated value and the measured value are shown for algorithm 1 (3c) and algorithm 2 (3d), it can be discovered that the calculated value overestimate more the higher the measured postfilter value is. This could suggest a systemic confounding factor, most likely in the mathematics predicting the citrate concentration in the patient.

As can be seen in Figs 2a, 2b and 3a–3d Algorithm 2 had a tendency to underestimate postfilter iCa compared to Algorithm 1 which was more balanced.

The correlation for Algorithm 1 is y = 0.13x + 0.36 and for Algorithm 2 y = 0.43x + 0.24 as is shown in Fig 3c and 3d. Ideally the correlation would be y = x. The correlation on a population basis was non-significant.

The Flexitrate regime aims at a BGA postfilter iCa range of 0.25–0.5 mmol/L and this interval can be considered as optimal. In three instances the BGA postfilter iCa were <0.25 mmol/L and in 35 instances the BGA postfilter iCa were >0.5 mmol/L. When the outliers were removed all the three postfilter iCa <0.25 mmol/L were left and 5 postfilter iCa >0.5 mmol/L were left. Both algorithms had issues detecting the five instances with too high postfilter iCa and therefore missed the intervention that should have followed. However, all the low postfilter iCa instances were detected. Of note, all instances with too high postfilter iCa occurred in treatments with a high postfilter iCa from the very beginning.

## Discussion

Both algorithms could predict a single unique value well with high trueness (mean difference) and precision (standard deviation) compared to a measured blood gas analysis as a reference, as can be seen in Table 2, but a population-based Pearson analysis did not show tight correlation.

Algorithm 1 showed better trueness and precision compared to Algorithm 2. Algorithm 1 did a better overall estimation compared to algorithm 2. Most likely a systemic confounding factor in the mathematics predicting the citrate concentration in the patient can be anticipated. To detect the confounding factor and correct it requires a large revision of the mathematics and testing of multiple real scenarios.

Algorithms always require input. The first algorithm used a total systemic calcium value from the laboratory as input, which in most cases was about 12 hours old, but up to 72 hours occurred.

Point of care devices are available that can measure many of the parameters used as input in the algorithms [14]. However, these are not continuous and additional devices are not desired in a busy scenario around an ICU patient. Most western ICUs possess a BGA and therefore our formulas were based on samples from the blood gas analyzer, or laboratory. The aim was to develop algorithms based on easily available input, and thus avoiding increasing the cost or workload for the ICU staff.

The patients’ starting levels varied, in some cases the starting point was stable within normal range, whereas in other cases ongoing shifts were present and most electrolyte balances were deteriorated. The calcium homeostasis is a central player and it is influenced by multiple functions in the body. The algorithms try to simulate and calculate a most probable scenario based on the input they get. However, in a complex disturbance in the body the algorithms may reach their limits. Too little input will increase the probability that the algorithms will not be able to predict the post-filter iCa with clinically acceptable precision and accuracy, and too much input will of course make the algorithms lose their importance.

Regression models are popular ways to find relations between substances in large data sets, but a major limitation is that the regression model obtained will be true only for the specific patient data used and not necessarily generalizable. Therefore, we wanted to create algorithms based on proven formulas from human physiology and biochemistry.

**Fig 4:**
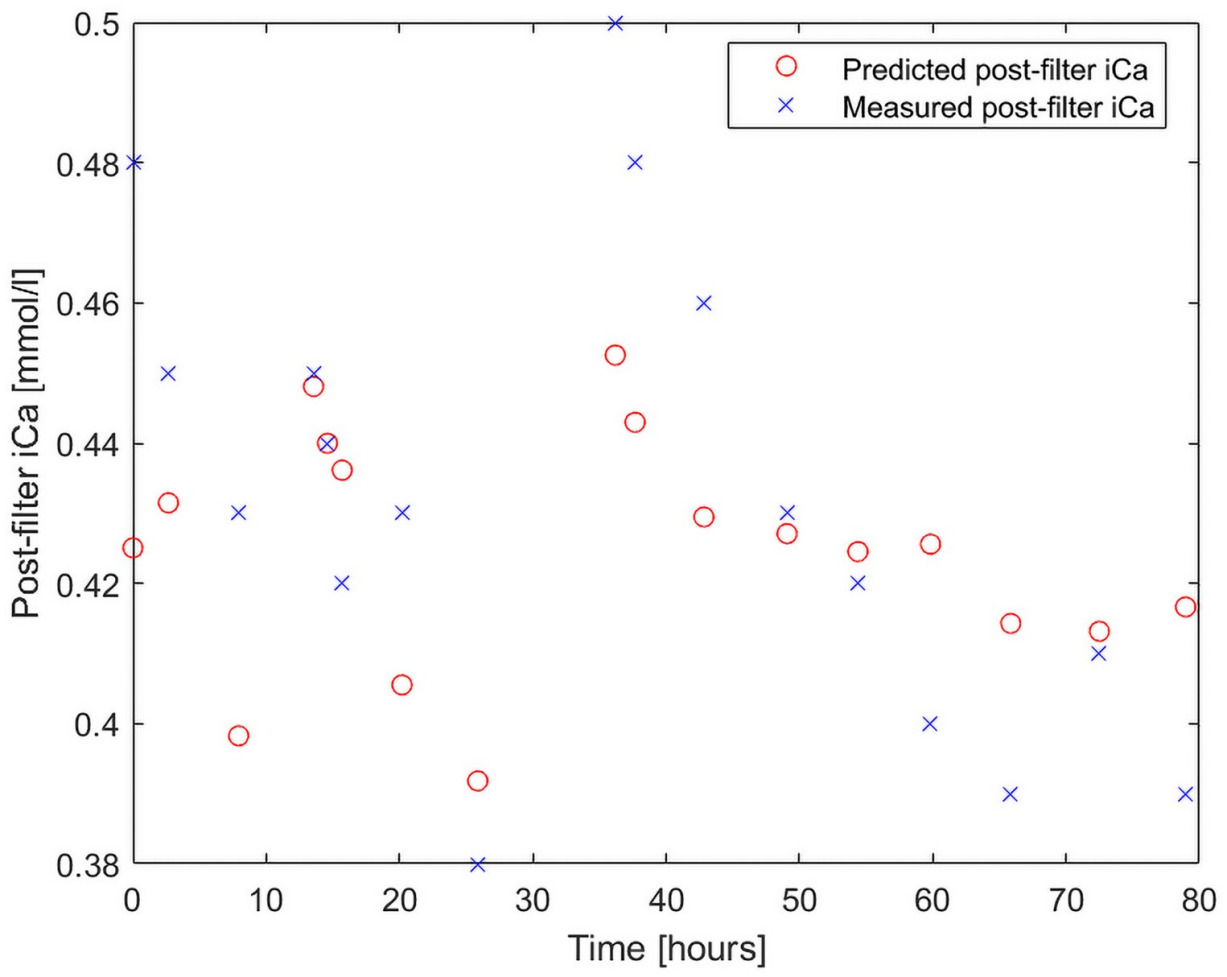
Measured versus calculated postfilter iCa values in a patient, with no obvious explanation on changes in measured BGA post-filter iCa values. There were no machine setting changes or changes in systemic iCa or other parameters that could explain the decrease in the BGA post-filter iCa over time (starting at 37th hour into the treatment and continuing to the 68th hour). Of note, the treatment was stopped at the 37th hour and restarted at the 39th hour, at a high postfilter iCa value. Most probably the pause in the treatment has an impact. What can also be noted is that the calculated values follow the decline.

The BGA showed greater distribution range compared to the predicted values, reflecting a large uncertainty in the BGA measurements [15]. Also, BGA values shifted without obvious explanations. Fig 4 shows alterations in BGA ionized calcium which cannot be explained by changes in machine settings nor by deranged systemic ionized calcium or total calcium levels. The BGAs are not approved for ionized calcium ranges 0.2–0.5 mmol/l, nor for samples with high citrate content. BGAs have a typical deviation range of ± 7.5% and the deviation can be larger in certain models [10, 11, 15]. Outliers can be a result of using the BGA outside the approved limits, but also of sampling errors. A previous study by *Schwarzer* [11] indicated that > 70% of the post-filter iCa samples could lead to incorrect therapy interventions depending on measurement errors of the BGA used [10–12, 15]. The inaccuracy of the BGAs and sample management could on a theoretical basis have an impact on the unfavorable correlation between the algorithm and BGA post-filter iCa values. There are unfortunately no alternative reference methods to BGAs available in hospitals today.

Initial assumptions were carried out by the algorithms without taking into account the existing citrate concentration in the patient nor the addition of citrate from the dialysis circuit. When we created a module in the algorithm to estimate the citrate’s impact a large improvement of the trueness and precision could be obtained [10]. However, we believe more work on the citrate module is still needed. Another improvement would be to increase the present resolution of one hour to as high as 5 minutes, since a stop of 18 minutes will lead to a large drop of citrate concentration in the patient because 50% of the citrate will be metabolized in this time [16]. The electronic medical record system works on hourly basis, but the black box on the CRRT machine has a resolution of seconds and offers a future possibility.

Mainly due to precision, the algorithms were not able to detect all instances where the BGA values would have implied an intervention. There are many reasons for this; in some scenarios the algorithm predicted values close to the intervention limit of 0.5 mmol/L, but on the wrong side compared to the BGA value.

The algorithms were worse in predicting high values, especially when the patient started CRRT with a high initial value. There were in total 5 instances when post-filter iCa measurements were > 0.5 mmol/L and only 3 instances when the value was < 0.25 mmol/L. These are too few in order to make assumptions on the trueness and precision of the algorithms compared to BGA.

On the contrary, values that did not lead to interventions were much greater in number and in those instances the algorithm was able to detection with high precision and trueness.

A small number of out-of-range values due to non-physiological discrepancies were observed and excluded. Unfortunately, the algorithms developed showed non-significant regression (seen in Fig 3a and 3b) and some of that can be due to the need to improve the citrate model, lack of machine data and the lack of having a great number of outliers as a control. The model is not able to perform at the precision needed to be able to replace a BGA at this time point.

In order to further develop the algorithms a better understanding of the citrate metabolism in critically ill patients is required. The citrate metabolism was predicted by using formulas from *Zheng* [9] and they need to be re-evaluated and elaborated. These formulas originate from stable patients with normal individual optimal level status [9].

Repeated safety checks on systemic iCa will always be required, but a more instant calcium handling within the CRRT circuit promoted by the algorithm will increase safety. A well working algorithm will have its place by increasing continuity and reducing the impact of human errors and clinical inexperience.

This set of two algorithms is to be considered as version 1 from which further development can take place with focus on detecting outliers more efficiently and to be adaptive in different calcium balance scenarios. In their present form the algorithms are able to predict values with high accuracy and precision for in-range values. A better understanding of the pivotal role of citrate metabolism is mandatory, and for that a higher time resolution of the extracted machine data will be needed.

## Conclusion

In conclusion, the algorithms were able to predict mean post-filter iCa with adequate trueness and precision for single values, but linear regression was not significant. Because the number of outliers was small significant assumptions are difficult to establish. Future aspects are that an algorithm which predicts the post-filter iCa could replace or work together with post-filter iCa measurements and enable continuous monitoring. However, work is still needed in order to make the algorithm more efficient and adaptive due to different calcium scenarios the patients present when entering the CRRT.

## Supporting information

S1 Fig (DOCX)

## Abbreviations

Acet_tot_: Total acetate [*mmol/L*]
Alb_tot_: Total albumin [*g/L*]
C(0): plasma citrate at t = 0 [*mmol/L*]
Ca_PD_: Total calcium post-dialyzer [*mmol/L*]
Ca_PW_: Total calcium in the plasma water post-dialyzer [*mmol/L*]
Ca_tot_: Total calcium [*mmol/L*]
C_bi_: Inlet blood concentration [*mmol/h*]
C_di_: Inlet dialysate concentration [*mmol/L*]
C_do_: Outlet dialysate concentration [*mmol/L*]
C_sys_: Systemic citrate concentation [*mmol/L*]
Cit_PD_: Total citrate post-dialyzer [*mmol/L*]
Cit_PW_: Total citrate in the plasma water post-dialyzer [*mmol/L*]
Cit_tot_: Total citrate [*mmol/L*]
Cl_tot_: Total chloride [*mmol/L*]
D_cit_: Citrate dose [*mmol/L of blood*]
F_CAL_: Fraction of patient plasma total calcium made availabe in plasma water for transfer through dialyzer membrane after citrate infusion [*Dimensionless*]
F_DIL_: Dilution ratio from plasma water reaching dialyzer [*Dimensionless*]
fpw: Plasma water fraction [*Dimensionless*]
fpw_PD_: Plasma water fraction post-dialyzer [*Dimensionless*]
G_met_: citrate load [*mmol/h*]
HCO_3tot_: Total bicarbonate [*mmol/L*]
Hct: Hematocrit [*Dimensionless*]
Hct_PD_: Hematocrit post-dialyzer [*Dimensionless*]
HPO_4tot_: Total phosphate [*mmol/L*]
iCa_ref_: Systemic ionized calcium reference level [*mmol/L*]
iCit: Systemic ionized calcium reference level in blood [*mmol/L*]
J_citload_: Citrate load in patient [*mmol/h*]
K: Filter clearance [*ml/h*]
K_Ca_: Plasma clearance of calcium in the dialyzer [*ml/h*]
K_CaB_: Equilibrium association constant for calcium bicarbonate [*mmol/L*]
K_CaCit_: Equilibrium association constant for calcium citrate [*mmol/L*]
K_CaCit2_: Equilibrium association constant for calcium dicitrate [*mmol/L*]
K_CaCO3_: Equilibrium association constant for calcium carbonate [*mmol/L*]
K_CaCO3_: Equilibrium association constant for calcium carbonate [*mmol/L*]
K_CaP_: Equilibrium association constant for calcium phosphate [*mmol/L]*
K_dialyzer_: Plasma clearance of citrate on the filter/dialyzer [*ml/h*]
K_patient_: Plasma clearance of citrate in the body [*ml/h*]
K_tot_: Total potassium [*mmol/L*]
Lact_tot_: Total lactate [*mmol/L*]
Mg_tot_: Total magnesium [*mmol/L*]
Na_tot_: Total sodium [*mmol/L*]
nCa_ref_: Number of sites that are occupied by calcium on a protein [*Dimensionless*]
Q_B_: Blood flow rate [*ml/min*]
Q_D_: Dialysate flow rate at dialysate inlet [*ml/h*]
Q_E_: Effluent flow rate [*ml/h*]
Q_fil_: Filtration flow rate [*ml/h*]
Q_PBP_: Flow of citrate-containing fluid [*ml/h*]
Q_PRE_: Flow rate of replacement fluid downstream of dialyzer [*ml/h*]
Q_pwinlet_: Plasma water flow rate at filter inlet [*ml/h*]
RT: Mass transfer resistance [*mmol/L*]
S: Effective surface area of the hemofilter/dialyzer [*m*^*2*^]
SC: Sieving coefficient of the membrane [*Dimensionless*]
SO_4tot_: Total sulphate [*mmol/L*]
t: Time [*h*]
V_d_: Volume of citrate distribution [*L*]
