# Supplementary material for: Mathematical modelling of post-filter ionized calcium during citrate anticoagulated continuous renal replacement therapy
Source: PLoS One. 2021 Feb 25;16(2):e0247477. doi: 10.1371/journal.pone.0247477 (PMC7906315; doi:10.1371/journal.pone.0247477)
Supplement: S1 Fig — (DOCX) [file pone.0247477.s001.docx]

**S1 Fig.**

Location 1 (Loc1) shows the spot, where the amount of removed calcium through the dialysis filter is calculated. The loss is used to set the calcium infusion rate (Q_SYR_).

Location 2 (Loc2) shows the spot, where post-filter iCa is calculated. The algorithm uses a blood model, which considers chemical properties.

$$Q_{PBP}$$

$$Q_{B}$$

$$Loc2$$

$$Q_{SYR}$$

$$Loc1$$

$$Q_{D}$$

$$Q_{E}$$
